# Immunotherapy with an antibody against CD1d modulates neuroinflammation in an α-synuclein transgenic model of Lewy body like disease

**DOI:** 10.1186/s12974-024-03087-7

**Published:** 2024-04-15

**Authors:** Michiyo Iba, Somin Kwon, Changyoun Kim, Marcell Szabo, Liam Horan-Portelance, Maria Lopez-Ocasio, Pradeep Dagur, Cassia Overk, Robert A. Rissman, Eliezer Masliah

**Affiliations:** 1 Laboratory of Neurogenetics, Molecular Neuropathology Unit, National Institute on Aging, National Institutes of Health, Bethesda, MD 20892 USA; 2 Flow Cytometry Core Facility, National Heart, Lung, and Blood Institute, National Institutes of Health, Bethesda, MD 20892 USA; 3 Department of Neurosciences, University of California, San Diego, La Jolla, CA 92093 USA; 4 Division of Neuroscience, National Institute on Aging, National Institutes of Health, Bethesda, MD 20814 USA

**Keywords:** α-synuclein, T cell, Parkinson’s disease, Dementia with Lewy bodies, Adaptive immunity, Innate immunity

## Abstract

The neuroinflammatory process in synucleinopathies of the aging population such as Parkinson’s disease (PD) and dementia with Lewy bodies (DLB) involves microglial activation as well as infiltration of the CNS by T cells and natural killer T cells (NKTs). To evaluate the potential of targeting NKT cells to modulate neuroinflammation, we treated α-syn transgenic (tg) mice (e.g.: Thy1 promoter line 61) with an antibody against CD1d, which is a glycoprotein expressed in antigen presenting cells (APCs). CD1d-presented lipid antigens activate NKT cells through the interaction with T cell receptor in NKTs, resulting in the production of cytokines. Thus, we hypothesized that blocking the APC-NKT interaction with an anti-CD1d antibody might reduce neuroinflammation and neurodegeneration in models of DLB/PD. Treatment with the anti-CD1d antibody did not have effects on CD3 (T cells), slightly decreased CD4 and increased CD8 lymphocytes in the mice. Moreover, double labeling studies showed that compared to control (IgG) treated α-syn tg mice, treatment with anti-CD1d decreased numbers of CD3/interferon γ (IFN γ)-positive cells, consistent with NKTs. Further double labeling studies showed that CD1d-positive cells co-localized with the astrocytes marker GFAP and that anti-CD1d antibody reduced this effect. While in control α-syn tg mice CD3 positive cells were near astrocytes, this was modified by the treatment with the CD1d antibody. By qPCR, levels of IFN γ, CCL4, and interleukin-6 were increased in the IgG treated α-syn tg mice. Treatment with CD1d antibody blunted this cytokine response that was associated with reduced astrocytosis and microgliosis in the CNS of the α-syn tg mice treated with CD1d antibody. Flow cytometric analysis of immune cells in α-syn tg mice revealed that CD1d-tet + T cells were also increased in the spleen of α-syn tg mice, which treatment with the CD1d antibody reduced. Reduced neuroinflammation in the anti-CD1d-treated α-syn tg mice was associated with amelioration of neurodegenerative pathology. These results suggest that reducing infiltration of NKT cells with an antibody against CD1d might be a potential therapeutical approach for DLB/PD.

**Supplementary Information:**

The online version contains supplementary material available at 10.1186/s12974-024-03087-7.

## Background

α-synuclein (α-syn), which is a neuronal protein [1] that is involved in modulating synaptic plasticity is also expressed in hematopoietic cells and plays a role in the development and maturation of B and T cells [2–4]. Consequently, in synucleinopathies of the aging population such as Parkinson’s disease (PD) and dementia with Lewy bodies (DLB) [5, 6], progressive accumulation of α-syn in neuronal cells in cortical [7, 8] and subcortical brain regions leads both to neurodegeneration of selective neuronal populations and immune dysregulation resulting in neuroinflammation [9]. This neuroinflammatory process involves activation of microglial cells with release of cytokines as well as infiltration by T cells in vulnerable brain regions [9].

Extracellular α-syn aggregates have been shown to activate microglia and astrocytes via toll like receptors (TLRs) to produce pro-inflammatory cytokines such as IL-1β, IL-6, and TNFα [10–13]. Moreover, since astrocytes might function as antigen presenting cells (APCs) in the CNS [14], it is possible that the propagating extracellular α-syn interacting with these cells might be partially responsible for activating the trafficking of T cells into the CNS. Moreover, astrocytes and microglial cells express major histocompatibility complex II (MHC-II), the T-cell inhibitory molecule PD-L1, and co-stimulatory markers CD40, CD80, and CD86 [14]. Together, these studies indicate that MHC-II^+^ microglia and astrocytes can directly activate CD4^+^ T cells, suggesting APC-like functionality. Peptides derived from α-syn have been shown to act as antigenic epitopes to drive helper and cytotoxic T cell responses in PD patients [15]. Moreover, recent studies have shown that in DLB and in α-syn tg models, T cells [16] migrate into the CNS via the CXCR4-CXCL12 axis that results in interleukin-17 production and neurotoxicity [17].

Natural killer (NK) and natural killer T (NKT) cells represent subsets of the innate immune system that are important for modulating the adaptive immune system [18]. The NKT cells are an unique subtype of CD1d restricted T cells with characteristics of NK- and T cells that can be subdivided into functional subsets [18]. NKT cells can switch between different functional subsets upon cell–cell interaction or interaction with signaling molecules [18]. In contrast, NK cells represent a population of specialized lymphocytes capable of recognizing and eliminating a wide range of cancer and virus-infected cells but not normal cells [18]. Even though the participation of T cells in neurodegenerative disease is well known, the understanding of role of NKT cells in neurodegeneration is limited [19]. Recent studies have shown that in addition to CD4 and CD8, α-syn also triggers migration of natural killer T (NKTs) and NK cells that are in close contact with astrocytes and microglia and result in the production of interferon (IFN) 𝛾, interleukin-6 (IL6) and other cytokines [16]. NKT cells co-express surface receptors characteristic of both T lymphocytes (e.g., CD3, α/β T-cell receptor [TCR]) and NK cells (e.g., CD56) [20], and so are postulated to act at the interface between the adaptive and innate immune systems [21]. Antigen recognition in NKT cells is restricted to those presented by the MHC-like molecule CD1d (cluster of differentiation 1) [22]. Recent studies have shown that NKT and NK cells are increased in number and more activated in early stages of PD [23]. These less studied lymphoid cellular populations might represent an important mode of regulating the interactions between the adaptive and innate immune response in DLB/PD and as an immunomodulatory target.

To investigate the potential of targeting NKT cells at regulating the neuroinflammatory process in DLB/PD models, we treated α-syn transgenic (tg) mice (e.g.: Thy1 promoter line 61) with an antibody against CD1d which is a glycoprotein expressed in antigen presenting cells (APCs). CD1d-presented lipid antigens activate NKT cells through the interaction with T cell receptor in NKTs resulting in the production of cytokines [24, 25]. Thus, we reasoned that blocking the APC-NKT interaction with an anti-CD1d antibody might reduce neuroinflammation and neurodegeneration in models of DLB/PD.

## Materials and methods

### Mice and anti-CD1d treatment

To investigate the effects of interfering with NKT trafficking in an α-syn tg model of DLB/PD, we performed behavioral, molecular, and neuropathological studies in 2 to 3-month-old (cohort 1) mice overexpressing human wild type α-synuclein (α-syn) under the Thy-1 promoter (Line 61, α-syn tg) and their non-transgenic (non-tg) littermate controls (cohort 1: *n* = 28, *n* = 14 male and 14 female). Both non-tg (*n* = 8) and α-syn tg mice (*n* = 6) were treated with 200 µg of anti-CD1d (Bio X Cell, CD1.1, clone number 20H2 [HB323]) or control IgG1 (Bio X Cell, rat IgG1, non-tg, *n* = 8; α-syn tg, *n* = 6) which was administered once a week for 3 months by intraperitoneal injection. For flow cytometry and additional molecular and neuropathological analysis a comparable second cohort of α-syn tg (*n* = 8 [*n* = 4 IgG, *n* = 4 anti-CD1d], *n* = 2 male and 6 female) mice and matched non-tg littermates (*n* = 8 [*n* = 4 IgG, *n* = 4 anti-CD1d], *n* = 6 male and 2 female) were prepared. We selected this particular model because α-syn tg mice of this age display considerable accumulation of α-syn in cortical and subcortical regions, degeneration of neurons in the deeper layers of the neocortex and limbic system, axonal degeneration in the striatonigral system, microglial and astrocytic activation, and release of interleukine-1β (IL-1β), IL-6, and tumor necrosis factor (TNFα) [26–29]. All mice used in this study were bred and analyzed at the National Institute on Aging (NIA) in the Bethesda campus.

### Tissue collection

All experiments were performed in accordance with protocols approved by the Institutional Animal Care and Use Committee of the NIA and institutional guidelines for the humane treatment of animals (463-LNG-2018, 463-LNG-2021). Sixteen of the first cohort mice were perfused with PBS and brains were divided for immunohistochemistry with paraffin processing, western blot and PCR, and twelve mice were non-perfused and used for vibratome sectioning, western blot and PCR. Before the perfusion of the second cohort mice, blood was collected from the animal’s heart, and all of the second cohort of mice were perfused and used for flow cytometry and immunohistochemistry with paraffin processing. The liver and the spleen were collected from the second cohort mice. We used the half hemisphere of the brain for IHC and the other one for biochemical analysis including western blotting and quantitative PCR experiments. We utilzed peripheral tissues and blood for FACS analysis. For immunohistochemical analysis, perfused mouse brains were fixed in 70% EtOH or 4% PFA and embedded in paraffin for serial sectioning at 6 μm with a microtome. Non-perfused mouse brains were fixed in 4% PFA for vibratome sectioning at 40 μm.

### Flow cytometry analysis

Blood sample was incubated with ACK Lysis Buffer for 5 min and stop reaction with FACS buffer. The suspension was pelleted by centrifugation and resuspended in FACS buffer for cell count. Spleen specimens were minced into smaller pieces and passed through a 40µM cell strainer with RPMI buffer, centrifuged and resuspended in ACK Lysis Buffer. The liver samples were also minced into smaller pieces and passed through a 70µM cell strainer with RPMI buffer, centrifuged and resuspended in 37% Percoll (GE Healthcare). Sample were then centrifuged and resuspended in ACK Lysis Buffer, and lysis reaction was stopped with FACS buffer. Cells were incubated with Fc Block (CD16/32, BD Biosciences, San Jose, CA), stained with antibodies for 30 min and fixed with 2% paraformaldehyde. Samples were acquired on the BD FACSSymphony A5 (BD Biosciences, CA) and analyzed using FlowJo (BD Biosciences, CA). Dead cells were excluded using the eBioscience Fixable Viability Dye eFluor® 506 (ThermoFisher Scientific, Waltham, MA). The following antibodies were used: anti-CD8 (740,278, BUV395), anti-CD4 (612,952, BUV496), anti-CD45 (748,370, BUV805), anti-NK 1.1 (560,618, APCcy7), anti-TCRγδ (553,178, PE) from BD biosciences, and anti-TCRβ (109,243, BV650) and anti-CD11b (101,206, BB515) from BioLegend (San Diego, CA). APC-conjugated mouse CD1d tetramers loaded with glycolipid PBS-57 (CD1d-tet) and an unloaded tetramer comprised of only the glycolipid PBS57 were obtained from the tetramer facility of the US National Institutes of Health.

### Gene expression analysis

Briefly as previously described [16], brains were disrupted and homogenized using a TissueRuptor II, and RNA was extracted from lysates using the RNeasy mini kit (Qiagen Venlo, Netherlands). DNA was eliminated from the samples by incubating with DNase (Qiagen). First strand cDNA synthesis was performed by using 1 µg of total RNA together with oligo(dT)_12–18_ and the Invitrogen SuperScript II Reverse Transcriptase (Thermo Fisher Scientific), according to the manufacturer’s instructions. Quantification of cytokine mRNA expression was conducted using quantitative real-time polymerase chain reaction (qPCR) performed on an Applied Biosystems ViiA™ 7 Real-Time PCR System (Thermo Fisher Scientific). Primers were obtained from Thermo fisher Scientific; Ifng (Mm01168134_m1), Ccl4 (Mm00443111_m1), Il6 (Mm00446190_m1), Il1β (Mm00434228_m1), Tnf (Mm00443258_m1), and β-actin (Mm00607939_s1). Quantification of gene expression was performed by the ΔΔCt method relative to β-actin [30].

### Immunohistochemistry, double labeling and image analysis

Paraffin (6 μm) slides of mouse brains were incubated overnight at 4 °C with primary antibodies: CD1d (Bio X Cell, BE0179, Rat monoclonal 1:1000), CD3 (abcam ab16669, rabbit monoclonal 1:100 Citrate buffer treatment, T cell marker), CD4 (abcam ab183685, rabbit monoclonal 1:1000 Tris buffer treatment, helper T cell marker), CD8 (abcam ab203035, rabbit polyclonal 1:500 Tris buffer treatment, suppressor T cell marker), CD20 (Invitrogen PA5-16701, rabbit polyclonal 1:500 citrate buffer treatment, B cell marker), glial fibrillary acidic protein (GFAP) (Millipore MAB3402, mouse monoclonal 1:1000, astrocytes marker), TMEM119 (abcam ab209064, rabbit polyclonal 1:1000 Citrate buffer treatment, microglial cell marker), Iba-1 (abcam ab178846, rabbit polyclonal 1:1000 Citrate buffer treatment, microglial cell marker), SYN-1 (BD Biosciences 610,787, mouse monoclonal 1:1000, ⍺-syn), phosphor-α-synuclein (81 A, generous gift from Drs. Trojanowski and Lee at the University of Pennsylvania, mouse monoclonal 1:20 K Citrate buffer treatment), NeuN (Millipore MAB377, mouse monoclonal 1:1000, neuronal marker) and TH (Millipore AB152, rabbit polyclonal 1:1000 Tris buffer treatment). Slides were then incubated in biotin-tagged anti-rabbit or anti-mouse or anti-rat or anti-goat IgG1 (1:400, Vector Lab) secondary antibodies, treated with Avidin DHRP (1:200, ABC Elite, Vector Lab), visualized with diaminobenzidine (DAB, Vector Lab), counterstained with hematoxylin, and imaged with Zeiss wide field microscope. For double immunolabeling, slides were incubated with the following antibody combinations: IFN𝛾 (R and D systems AF-585, goat polyclonal 1:200)/CD3 (red, rabbit); CD1d (green, rat)/CD3 (red, rabbit); CD1d (red, rat)/GFAP (green, mouse) and CD3 (red, rabbit)/GFAP (green, mouse). For each combination, markers were visualized with FITC-tagged and Texas-red secondary antibodies, respectively. Nuclei were stained with DAPI (Hoechst 33,258), and the slides mounted under glass coverslips with anti-fading media (Vector Lab).

All slides were processed and imaged under the same standardized conditions and blind coded. Four fields from the frontal cortex, hippocampus, striatum, and thalamus were examined for each section and performed in duplicate for each mouse. Sections visualized with DAB were scanned with a Hamamatsu digital slide scanner and analyzed with an image program to determine the number of CD3, CD4, CD8, GFAP, and Iba-1 positive cells per field (230 μm × 184 μm). Double immunolabeled slides were imaged with an Apotome II mounted in a Carl Zeiss AxioImager Z1 microscope. Double labeled images were analyzed via the Zen 2.3 platform to determine de co-localization between CD3 positive cells displaying IFN𝛾 immunoreactivity and GFAP positive cells displaying CD1d immunostaining. Double labeled images were also used to determine the average proximity of CD3 cells to astrocytes displaying GFAP or CD1d immunolabeling.

### Protein extraction and western blot analysis

The hemibrains of animals excluding the olfactory bulb and cerebellum were sonicated with TBS buffer (4 × volume) containing protease inhibitor (PI) and phosphatase inhibitor (PPI) (1:100 each) and stored in 100 µl aliquot at −80 °C until use. 100 µl of PDGF buffer (HEPES 1mM, Benzamidine 5mM, 2-mercaptoethanol 2 mM, EDTA 3 mM, Magnesium Sulfate 0.5 mM, Sodium Aide 0.05%, pH 8.8) with PI and PPI (1:100 each) were added to 100 µl of TBS homogenate, and sonicated and centrifuged for 5 min at 5000*g* at 4 °C. Supernatant was collected and centrifuged for 60 min at 100,000*g* at 4 °C, and the supernatant was collected as the cytosolic fraction. The pellet was re-suspended with 40 µl of PDGF buffer, sonicated and saved as the particulate fraction. 20 µg of total protein was loaded for western blotting. The proteins were separated by electrophoresis with 0–12% gel (Bio-Rad, Hercules, CA), and transferred to PVDF membranes using semi-dry Trans-Blot Turbo Transfer System (Bio-Rad). Membranes were blocked with Odyssey blocking buffer (LiCor Biosciences, Lincoln, NE) and probed with primary and followed by fluorescence-tagged secondary antibody. The fluorescent signal detection and densitometric analysis were performed using ODAYSSEY CLx and Image Studio (LiCor Biosciences).

### Statistical analysis

Values shown in the figures are presented as mean ± SEM. P-values for determination of the statistical significance of differences were calculated using two-way ANOVA with post-hoc Tukey multiple comparison tests with Prism 10 (GraphPad, Boston, MA).

## Results

### Effects of anti-CD1d immunotherapy on T and NKT cells trafficking into the CNS of α-syn tg mice

**Fig. 1:**
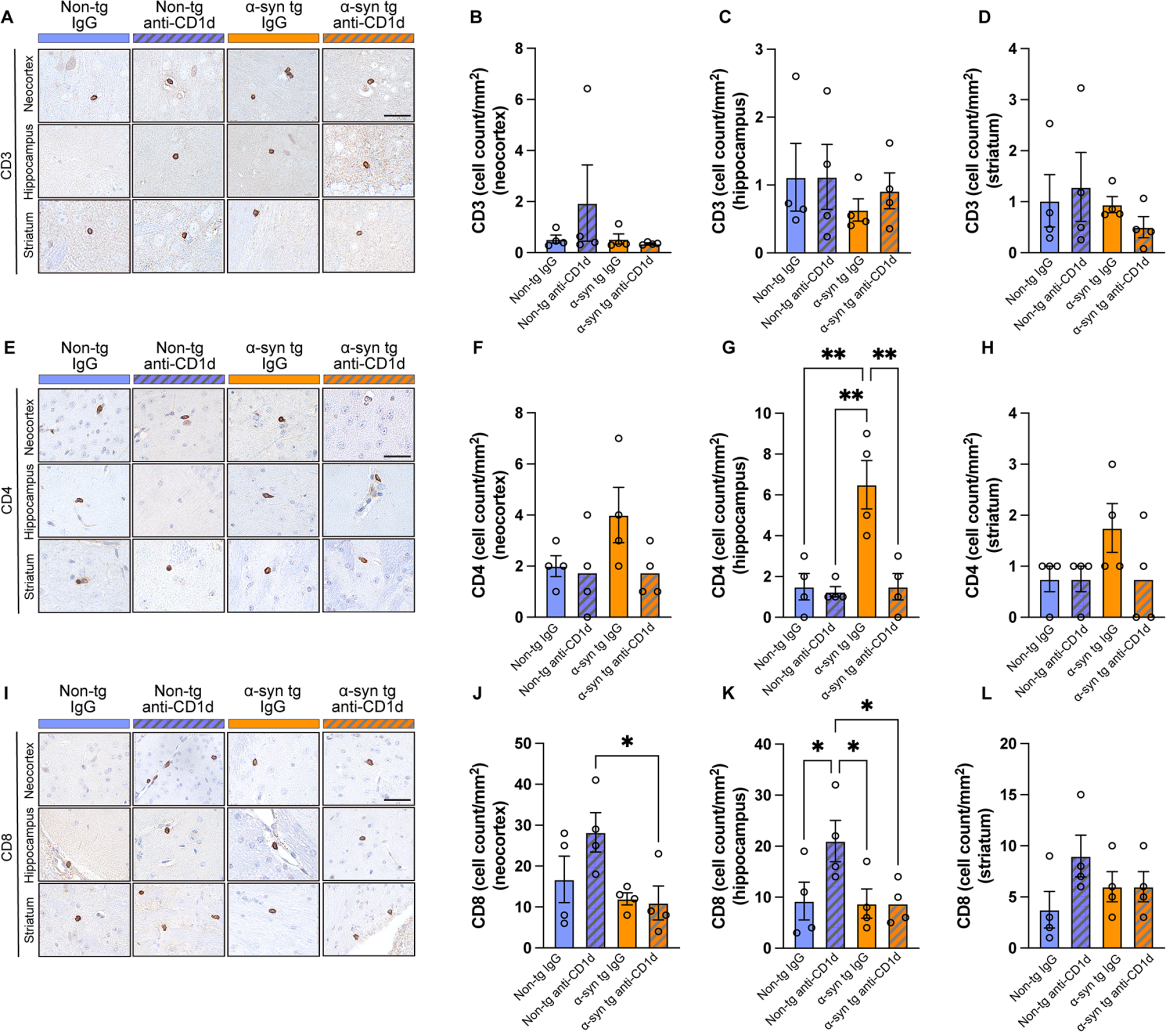
Immunohistochemical analysis of lymphoid cells in the brains of ⍺-synuclein transgenic mice treated with anti-CD1d. (**A**) Representative bright field light microscopy images from the neocortex, hippocampus and striatum of non-tg and ⍺-syn tg mice treated with IgG control or anti-CD1d and immunostained with an antibody against CD3 (general T cell marker). (**B-D**) Computer based image analysis showing comparable numbers of CD3 positive cell numbers in the neocortex (**B**), hippocampus (**C**) and striatum (**D**) of non-tg and ⍺-syn tg treated mice. (**E**) Representative bright field light microscopy images from the neocortex, hippocampus and striatum of non-tg and ⍺-syn tg mice immunostained with an antibody against CD4 (helper T cell marker). (**F-H**) Computer based image analysis showing increase numbers of CD4 positive cell in the brains of IgG injected ⍺-syn tg mice and lower levels in CD1d treated mice. (**I**) Representative bright field light microscopy images from the neocortex, hippocampus, and striatum of non-tg and ⍺-syn tg mice immunostained with an antibody against CD8 (cytotoxic T cell marker). (**J-L**) Computer based image analysis showing increase of CD8 positive cells in non-tg and ⍺-syn tg mice treated with anti-CD1d. Scale bar = 20 μm (high magnification). *n* = 4 mice per group. Statistical significance determined by two-way ANOVA with post-hoc Tukey; *, *p* ≤ 0.05; **, *p* ≤ 0.01

**Fig. 2:**
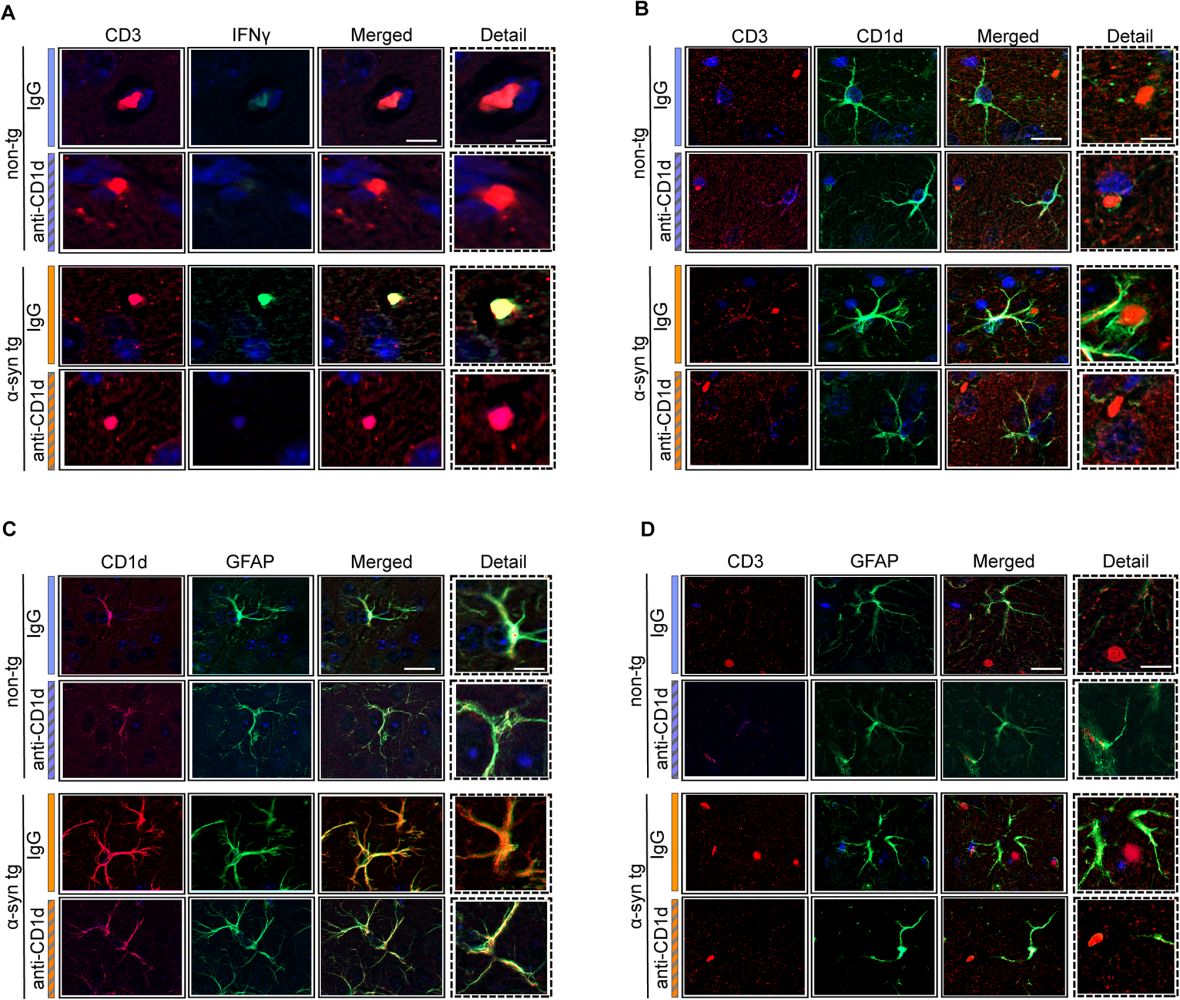
Double immunohistochemical analysis of NKT cells in the brains of ⍺-synuclein transgenic mice. (**A**) Split and merged representative microscopy images from the neocortex of non-tg and ⍺-syn tg mice treated with IgG or anti-CD1d double labeled with antibodies against CD3 (red channel) and IFN𝛾 (FITC channel), co-localizing CD3 and IFN𝛾 (merged images, yellow) suggests representing of NKT cells. (**B**) Split and merged representative microscopy images from the neocortex of non-tg and ⍺-syn tg mice treated with IgG or anti-CD1d double labeled with antibodies against CD3 (red channel) and CD1d (FITC channel), showing the relative proximity of T cells to the processes of glial cells expressing CD1d. (**C**) Split and merged representative microscopy images from the neocortex of non-tg and ⍺-syn tg mice treated with IgG or anti-CD1d double labeled with antibodies against CD1d (red channel) and GFAP (FITC channel). (**D**) Split and merged representative microscopy images from the neocortex of non-tg and ⍺-syn tg mice treated with IgG or anti-CD1d double labeled with antibodies against CD3 (red channel) and GFAP (FITC channel), showing the relative proximity of T cells to the processes of glial cells expressing GFAP. Scale bars = 15 μm (low magnification) and 5 μm (high magnification)

We have previously shown that the neuroinflammatory process in DLB/PD includes infiltration by T cells with a profile of CD4 and NKT cells [16]. To ascertain the effects of blocking NKT cells on neuroinflammation, we treated α-syn tg and non-tg mice with an antibody against CD1d and control IgG, and evaluated by immunohistochemical analysis with CD3 (total T cells), CD4 (helper T cells), CD8 (cytotoxic T cells), and CD20 (B cells) lymphocytes and by double immunolabeling with CD3/IFN𝛾, suggestive of NKT cells. Analysis of overall number of T cells with the CD3 antibody showed variable numbers of positive cells in the non-tg and α-syn tg mice treated with IgG or anti-CD1d with no significant differences (Fig. 1A–D). The CD4-positive T cells were slightly increased when comparing IgG treated non-tg to α-syn tg, and treatment with anti-CD1d brought levels of CD4 down to baseline in the α-syn tg (Fig. 1E–H). In contrast, compared to control IgG, non-tg mice treated with anti-CD1d displayed an increase of CD8 positive T cells, and in the α-syn tg mice treated with CD1d there was a mild increase in CD8 cells that was not significant (Fig. 1I–L). Very few CD20-positive B cells were detected with no differences among groups (not shown). To ascertain the effects of the antibody therapy on lymphocytes consistent with NKTs, brain sections were double labeled with antibodies CD3/IFNγ. In the non-tg mice, most cells were only CD3-positive and rare or none CD3/IFNγ-positive (Fig. 2A). The IgG treated α-syn tg mice showed the presence of CD3/IFNγ-positive cells while α-syn tg immunized with the antibody against CD1d displayed decreased numbers of CD3/IFNγ-positive cells (Fig. 2A).

**Fig. 3:**
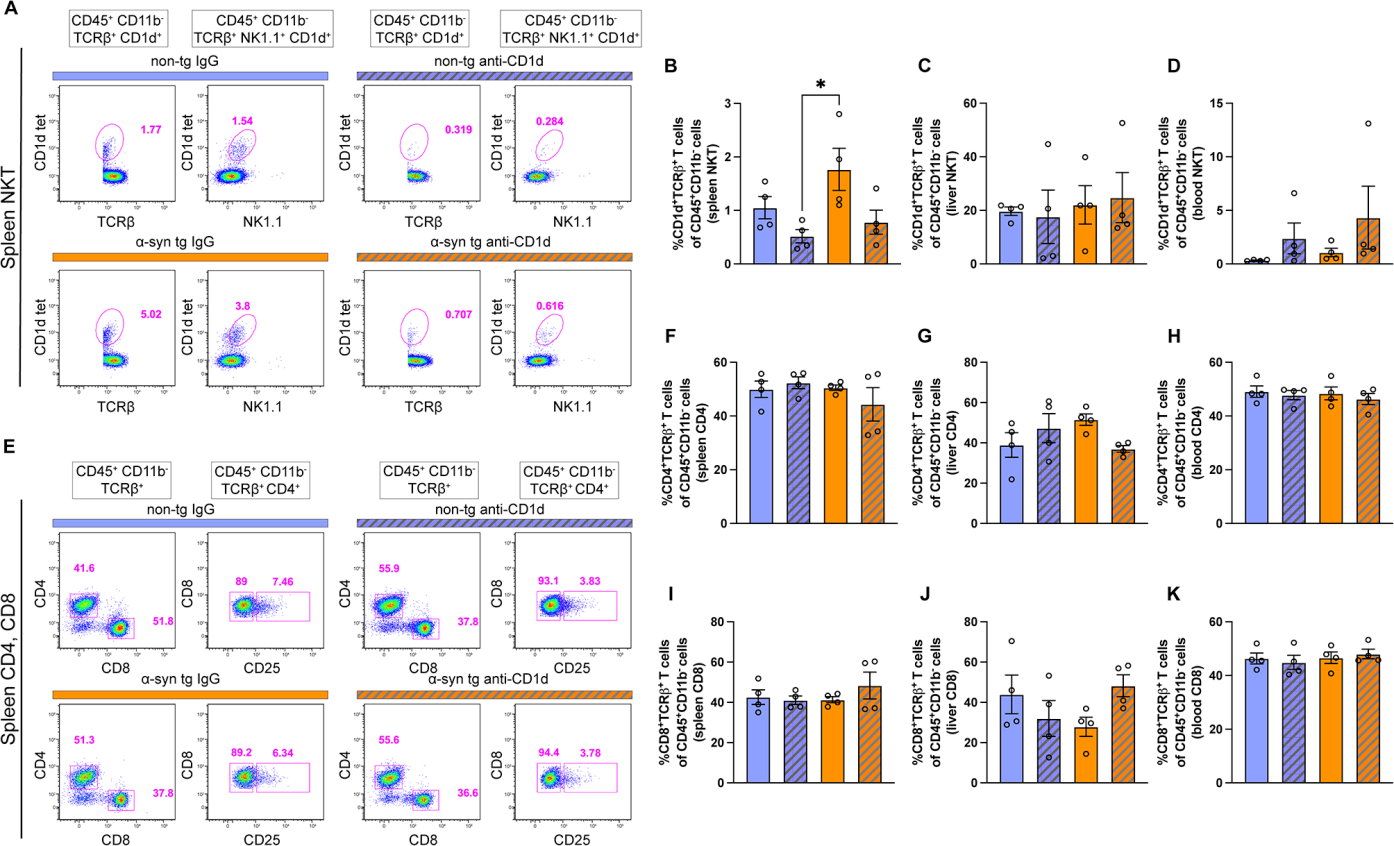
Flow cytometry analysis of T cells in blood and peripheral tissues of ⍺-synuclein transgenic mice treated with CD1d. (**A**) Representative flow cytometry plots of CD1d-tet and TCRβ^+^ expression in the spleen; (**B**–**D**) graphs of the frequency of NKT cells of CD45^+^CD11b^−^ cells in the spleen, liver and blood of non-tg and ⍺-syn tg mice treated with IgG or anti-CD1d. (**E**) Representative flow cytometry plots of TCRβ^+^ CD45^+^ expression in spleen; (**F**–**H**) graphs of frequency of CD4^+^ cells in the spleen, liver and blood respectively and (**I**–**K**) CD8^+^ cells in the spleen, liver and blood respectively. *n* = 4 mice per group. Statistical significance determined by two-way ANOVA with post-hoc Tukey; *, *p* ≤ 0.05

Flow cytometric analysis of immune cells showed that the CD1d-tet + T cells (consistent with NKTs) were elevated in the spleen of IgG treated α-syn tg mice compared to IgG control non-tg mice and treatment with the CD1d antibody reduced the cell numbers (Fig. 3A, B), but, no differences among groups were observed in liver or blood (Fig. 3C, D). Likewise, analysis of CD4 and CD8 cells by flow cytometry showed similar numbers in the spleen, liver, and blood among the four groups (Fig. 3E–K). The complete gating strategy of the spleen, liver, and blood is presented in Supplementary Figs. S1–S3, representative images of the flow cytometry for CD4 and CD8 cells in liver and blood are presented in Supplementary Fig. S4.

In summary, this part of the study showed that immunotherapy with anti-CD1d might have some effects on modulating the trafficking of CD4/CD8 and NKT cells in the α-syn tg model.

### Effects of anti-CD1d immunotherapy on APCs and neuroinflammation in α-syn tg mice

Previous studies have shown that astrocytes might play a role as antigen presenting cells in the CNS [14, 31]. To evaluate the effects of the CD1d immunotherapy on astrocytes and their interactions with T cells, we performed double labeling experiments. In the non-tg mice, CD3 positive T cells were distant to the processes of CD1d positive cells for IgG or anti-CD1d treatment (Fig. 2B). In contrast, in the IgG treated α-syn tg mice, T cells were closer to CD1d positive cells, whilst the T cells were distant to the process of the CD1d positive cells (Fig. 2B) in α-syn tg mice treated with anti-CD1d. Similarly, in the non-tg mice, occasional GFAP positive astrocytes displayed mild CD1d positivity (Fig. 2C). While more abundant colocalization between GFAP and CD1d was observed in the IgG treated α-syn tg mice, the GFAP positive astrocytes showed decreased levels of colocalization with CD1d immunolabeling in the anti-CD1d treated α-syn tg (Fig. 2C). In the non-tg mice, CD3 positive T cells were distant to the processes of astrocytes (Fig. 2D). In contrast, in the IgG treated α-syn tg mice, T cells were closer to astrocytes, whilst in α-syn tg mice treated with anti-CD1d, the T cells were distant to the process of the astrocytes (Fig. 2D).

**Fig. 4:**
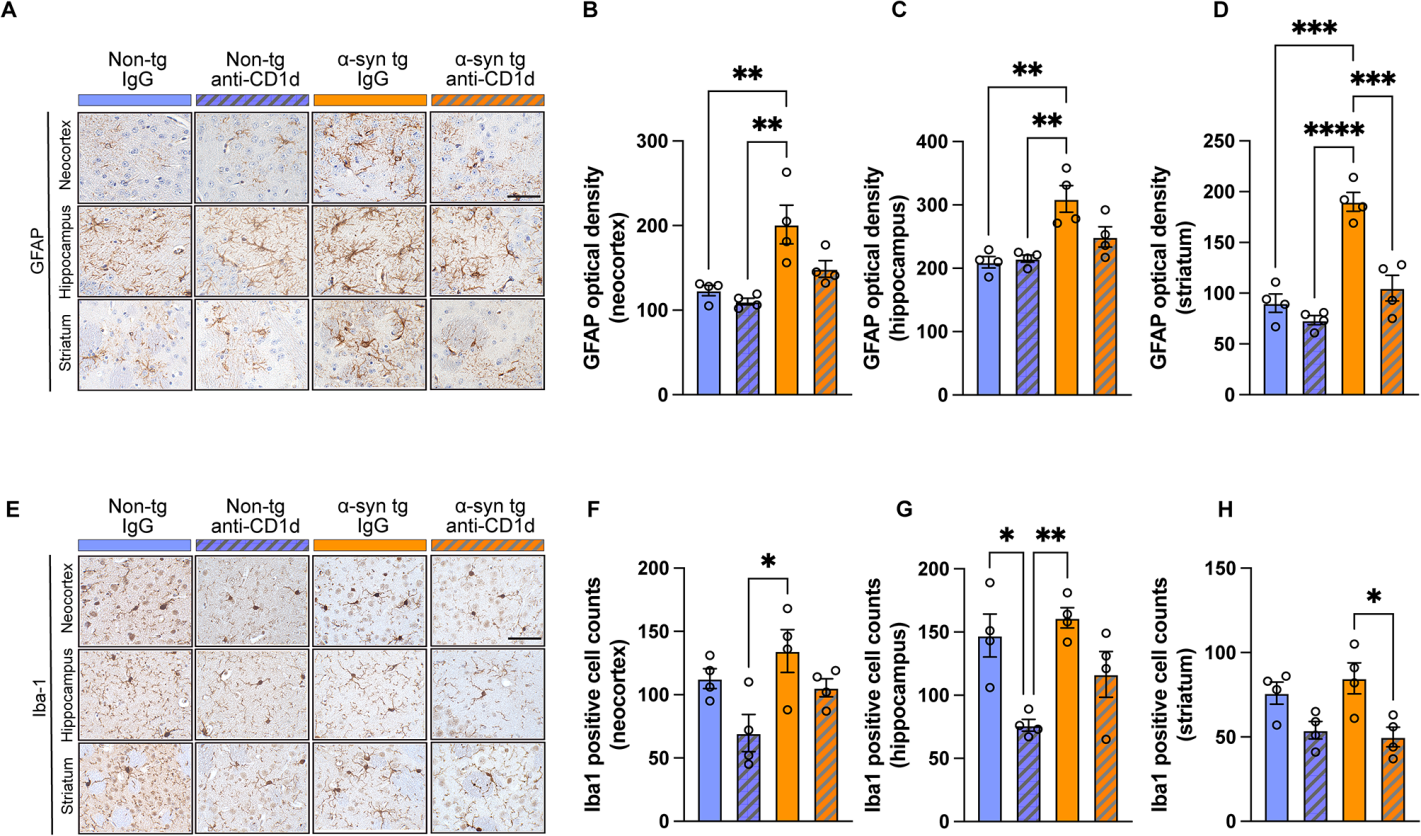
Immunohistochemical analysis of astrocytes and microglial cells in the brains of ⍺-synuclein transgenic mice treated with anti-CD1d. (**A**) Representative bright field light microscopy images from the neocortex, hippocampus, and striatum of non-tg and ⍺-syn tg mice treated with IgG or anti-CD1d immunostained with an antibody against GFAP (astrocytes marker). (**B**–**D**) Computer based image analysis showing increased GFAP immunoreactivity in the neocortex, hippocampus, and striatum of ⍺-syn tg mice and decreased levels with anti-CD1d treatment. (**E**) Representative bright field light microscopy images from the neocortex, hippocampus, and striatum of non-tg and ⍺-syn tg mice treated with IgG and anti-CD1d immunostained with an antibody against Iba-1 (microglial cell marker). (**F**–**H**) Computer based image analysis showing increased numbers of Iba-1 positive cells in the neocortex, hippocampus, and striatum in IgG and anti-CD1d treated non-tg and ⍺-syn tg mice. Scale bar = 50 μm (high magnification). *n* = 4 mice per group. Statistical significance determined by two-way ANOVA with post-hoc Tukey; *, *p* ≤ 0.05; **, *p* ≤ 0.01; ***, *p* ≤ 0.001; ****, *p* ≤ 0.0001

**Fig. 5:**
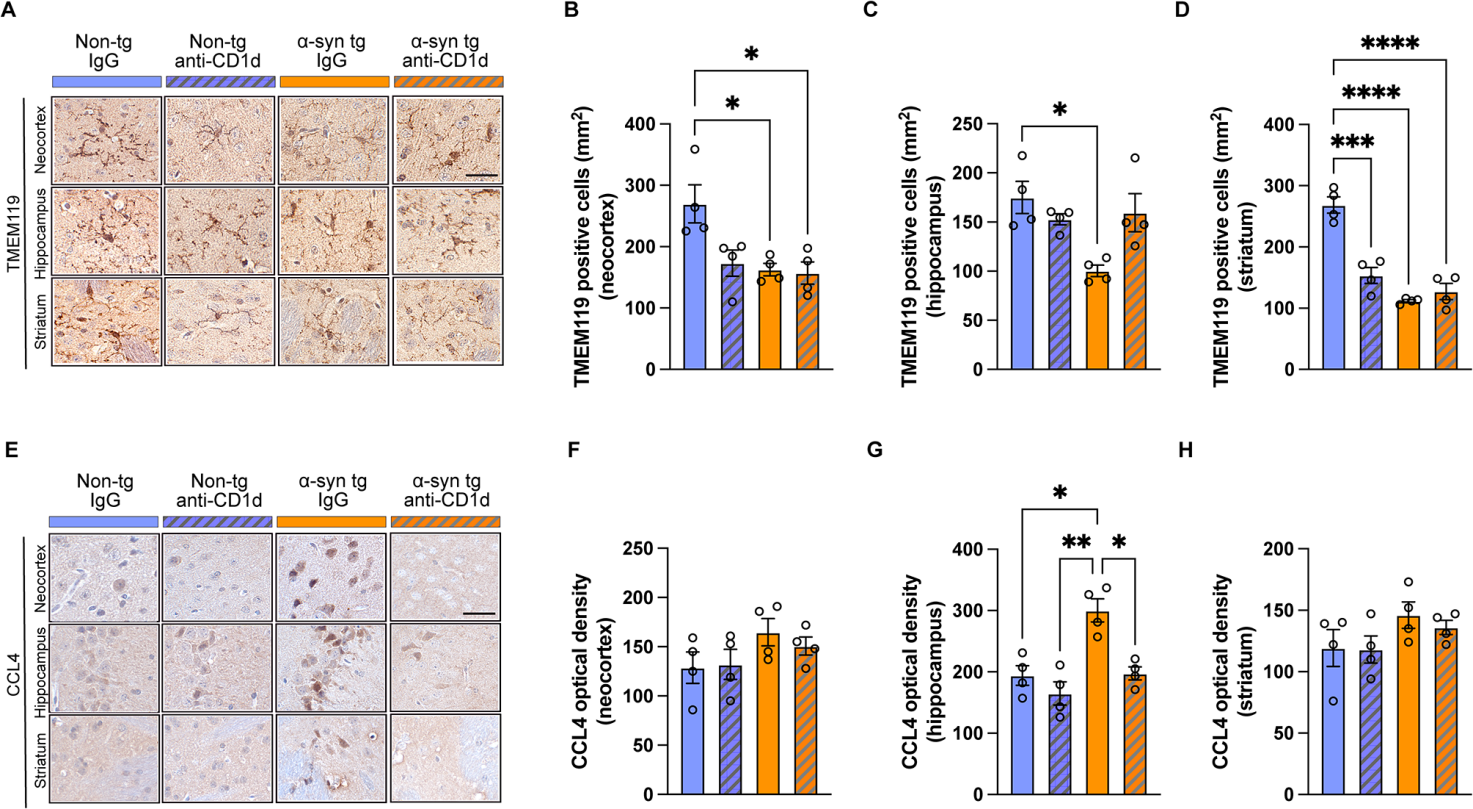
Immunohistochemical analysis of microglial activation in the brains of ⍺-synuclein transgenic mice treated with anti-CD1d. (**A**) Representative bright field light microscopy images from the neocortex, hippocampus, and striatum of non-tg and ⍺-syn tg mice treated with IgG or anti-CD1d immunostained with an antibody against TMEM119 (microglia cell marker). (**B**–**D**) Computer based image analysis showing the levels of TMEM119 immunoreactivity in the neocortex, hippocampus and striatum in IgG and anti-CD1d treated non-tg and ⍺-syn tg mice. (**E**) Representative bright field light microscopy images from the neocortex, hippocampus, and striatum of non-tg and ⍺-syn tg mice treated with IgG or anti-CD1d immunostained with an antibody against CCL4 (cell marker). (**F**–**H**) Computer based image analysis showing the levels of CCL4 immunoreactivity in the neocortex, hippocampus, and striatum in IgG and anti-CD1d treated non-tg and ⍺-syn tg mice. Scale bar = 10 μm (high magnification). *n* = 4 mice per group. Statistical significance determined by two-way ANOVA with post-hoc Tukey; *, *p* ≤ 0.05; **, *p* ≤ 0.01; ***, *p* ≤ 0.001; ****, *p* ≤ 0.0001

In agreement with these results, single labeling analysis of brain sections with the GFAP antibody showed increased astrocytosis in the neocortex, hippocampus, and striatum of the α-syn tg treated with IgG compared to the non-tg mice (Fig. 4A–D). Treatment with anti-CD1d reduced the levels of astrocytosis in the α-syn tg mice (Fig. 4A–D). Analysis of general populations of microglial cells with an antibody against Iba-1 showed that the anti-CD1d treatment to both non-tg and α-syn tg mice reduced microglial cells in the neocortex, hippocampus and striatum (Fig. 4E-H), but the statistical analysis showed that the significance was detected only non-tg mice hippocampus and α-syn tg mice striatum between IgG and anti-CD1d treatment (Fig. 4G, H). In contrast, analysis of homeostatic microglia with an antibody against TMEM119 showed decreased microglia immunostaining in the neocortex, hippocampus and striatum of the IgG treated α-syn tg compared to the non-tg mice (Fig. 5A–D). Treatment with anti-CD1d increased the number of TMEM119 positive microglia in the α-syn tg mice in the hippocampus (Fig. 5A, C), but no reduction or increase were shown in the neocortex (Fig. 5A, B) and striatum (Fig. 5A, D). We observed decreased numbers of TMEM119 positive microglia in non-tg mice treated with anti-CD1d (Fig. 5B–D). Since trafficking of T cells to the CNS might also be mediated by CCL4 [32], we analyzed this marker by immunohistochemistry and PCR in the mouse brains. Mild CCL4 expression was observed mostly in neuronal cells and few glial cells in the non-tg mice, but the levels of CCL4 expression were increased in the hippocampus (Fig. 5E, G) and to a lesser extent in the neocortex and striatum in the IgG treated α-syn tg mice (Fig. 5E, F, H). Treatment with anti-CD1d reduced CCL4 immunoreactivity in the α-syn tg to the level similar to non-tg mice (Fig. 5E–H).

**Fig. 6:**
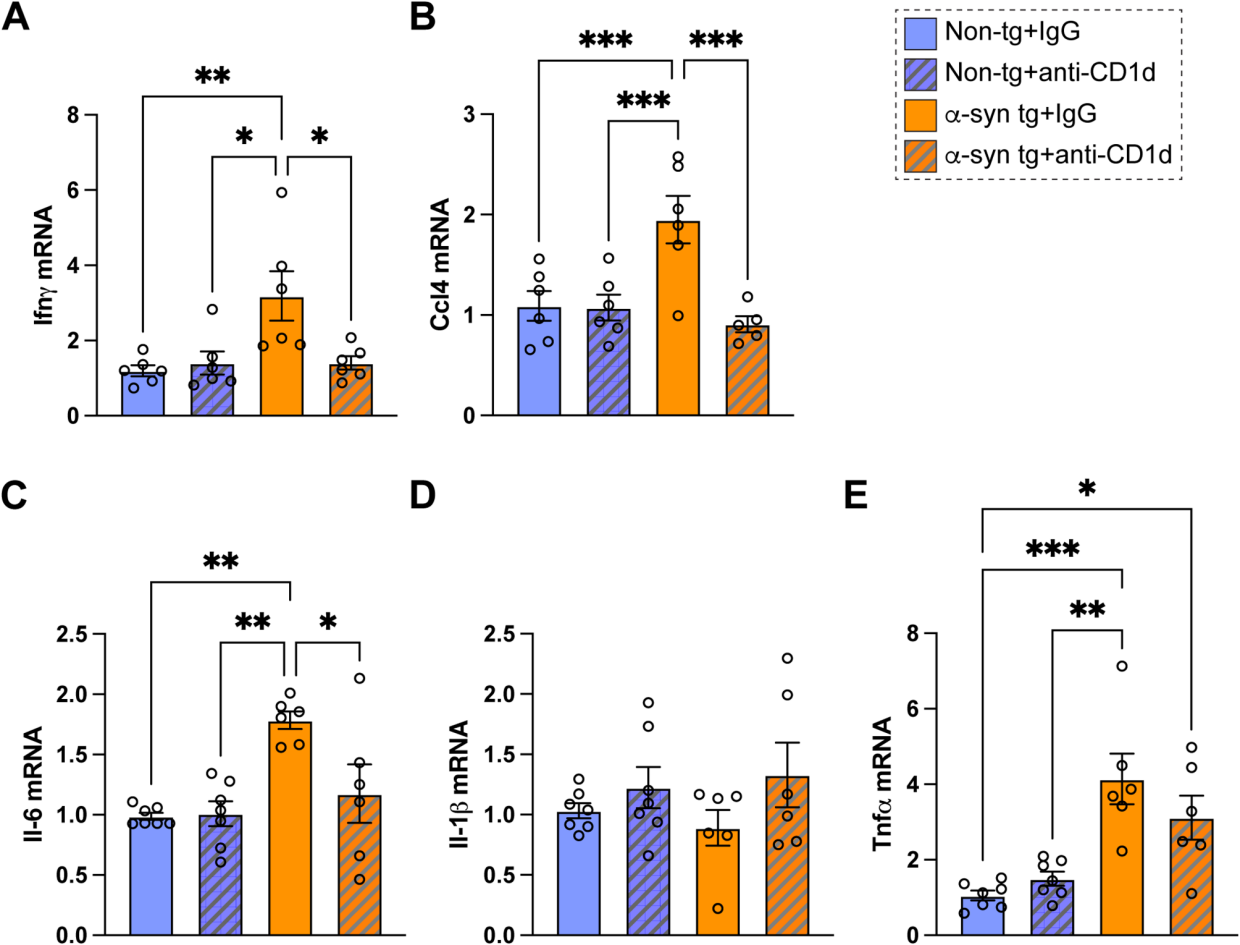
Transcriptomics analysis of cytokines and chemokines in the brain of α-syn tg mice treated with anti-CD1d. Expressions of (**A**) IFNγ, (**B**) CCL4, (**C**) IL-6, (**D**) IL-1β, and (**E**) TNFα, were determined by qPCR. *n* = 4 mice per group. Statistical significance determined by two-way ANOVA with post-hoc Tukey; *, *p* ≤ 0.05; **, *p* ≤ 0.01; ***, *p* ≤ 0.001

Consistent with these results, quantitative PCR analysis showed that levels of IFN𝛾, CCL4, and IL6 were increased in the brains of IgG treated α-syn tg mice compared to non-tg (Fig. 6A–C), whereas levels in the α-syn tg mice were comparable to the non-tg when treated with the anti-CD1d antibody (Fig. 6A–C). The expression of IL1β was not affected by genotype or treatment (Fig. 6D), while the level of TNFα was increased in the brains of IgG treated α-syn tg mice compared to non-tg, and treatment with the anti-CD1d antibody showed a trend toward decreased levels in α-syn tg mice (Fig. 6E).

Taken together, these results suggest that blocking the interactions between NKTs and glial cells with the antibody against CD1d might reduce inflammation in models of synucleinopathy.

### Effects of anti-CD1d immunotherapy on α-syn accumulation and neurodegeneration in tg mice

**Fig. 7:**
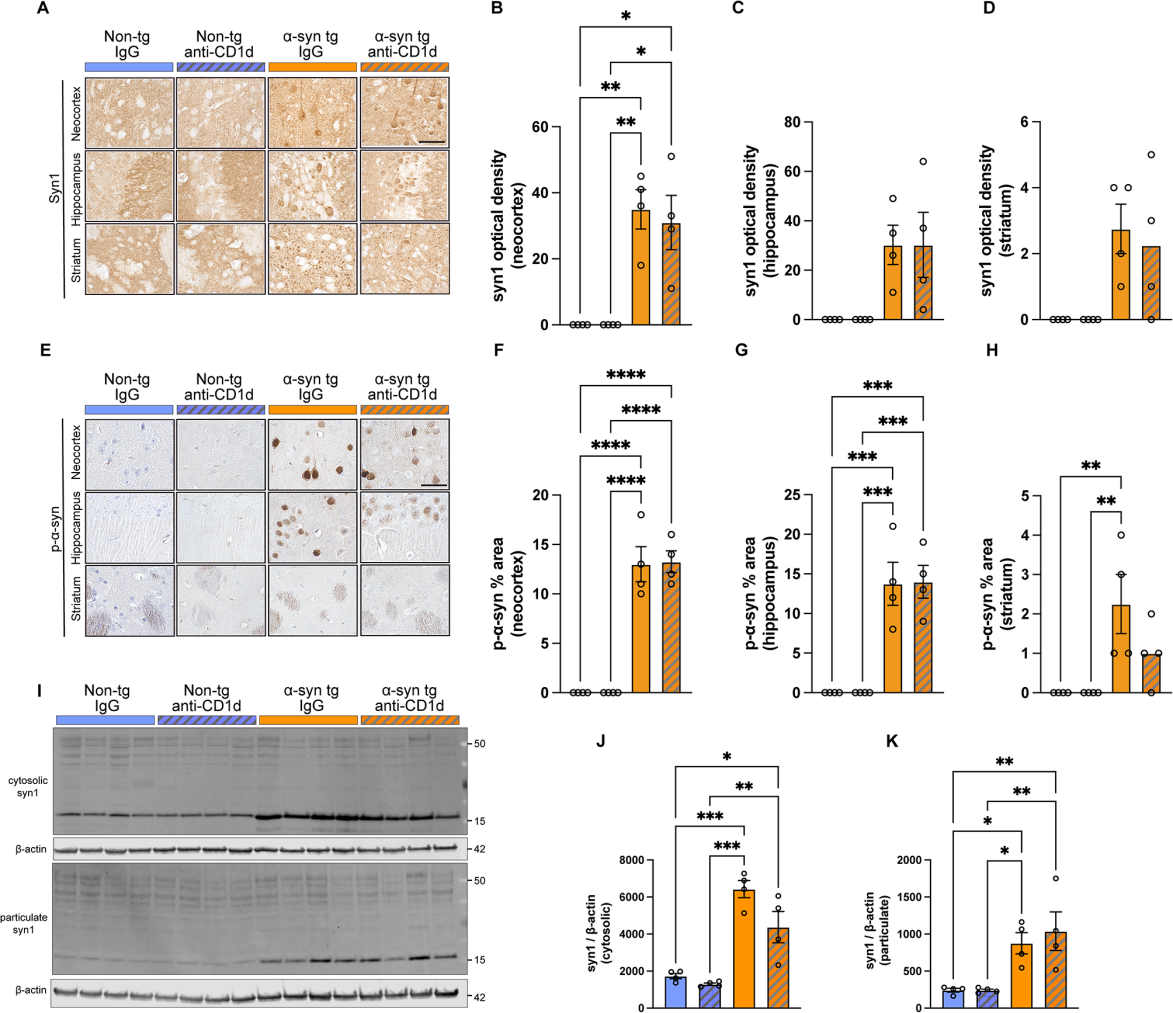
Immunochemical analysis of ⍺-synuclein accumulation in the brains of ⍺-synuclein transgenic mice treated with anti-CD1d. (**A**) Representative bright field light microscopy images from the neocortex, hippocampus, and striatum of non-tg and ⍺-syn tg mice treated with IgG or anti-CD1d immunostained with an antibody against total ⍺-syn. (**B**–**D**) Computer based image analysis showing comparable levels of total ⍺-syn immunoreactivity in the neocortex, hippocampus, and striatum of IgG or anti-CD1d treated ⍺-syn tg mice. (**E**) Representative bright field light microscopy images from the neocortex, hippocampus, and striatum of non-tg and ⍺-syn tg mice treated with IgG or anti-CD1d immunostained with an antibody against p129 ⍺-syn. (**F**–**H**) Computer based image analysis showing similar levels of p129 ⍺-syn immunoreactivity in the neocortex, hippocampus, and striatum in IgG or anti-CD1d treated ⍺-syn tg mice. (**I**) Representative western blot analysis of brain homogenates from cytosolic and particulate fractions of non-tg and ⍺-syn tg mice treated with IgG or anti-CD1d. (**J**, **K**) Computer based image analysis showing slight reduction in total ⍺-syn in the cytosolic fraction of ⍺-syn tg mice treated with anti-CD1d but not in the particulate fraction. Scale bar = 30 μm. *n* = 4 mice per group. Statistical significance determined by two-way ANOVA with post-hoc Tukey; *, *p* ≤ 0.05; **, *p* ≤ 0.01; ***, *p* ≤ 0.001; ****, *p* ≤ 0.0001

Next, we investigated the effects of reducing the interactions between NKTs and glial cells with anti-CD1d on α-syn aggregates. Immunohistochemical analysis with an antibody against total α-syn showed as expected in the non-tg IgG and anti-CD1d groups the same pattern and levels of immunoreactivity in the neuropil in the neocortex, hippocampus and striatum, while in the α-syn tg mice increased immunostaining was observed in the neuropil and neuronal cells bodies in all brain regions, and α-syn tg mice treated with anti-CD1d showed a mild decrease of α-syn immunoreactivity, but it was not significant (Fig. 7A–D). Immunohistochemical analysis with an antibody against phosphor-S129 α-syn showed no reactivity in the brains of the non-tg mice, in contrast, in the α-syn tg mice, there was intense neuronal accumulation of p-α-syn in the neocortex and hippocampus, although no differences were detected between the IgG and anti-CD1d treated groups (Fig. 7E–G) except a slight effect in the striatum (Fig. 7E, H). In agreement with the immunocytochemical analysis, the western blot analysis showed comparable low levels of expression of α-syn monomer in the cytosolic fraction in non-tg mice, with increase α-syn levels in the IgG α-syn tg mice with a slight but not significant decrease in the anti-CD1d group compared to the IgG (Fig. 7I, J). Similarly in the particulate fraction low levels of α-syn were detected in non-tg and increased in the α-syn tg mice, with no differences between the IgG and anti-CD1d treated groups (Fig. 7I, K).

**Fig. 8:**
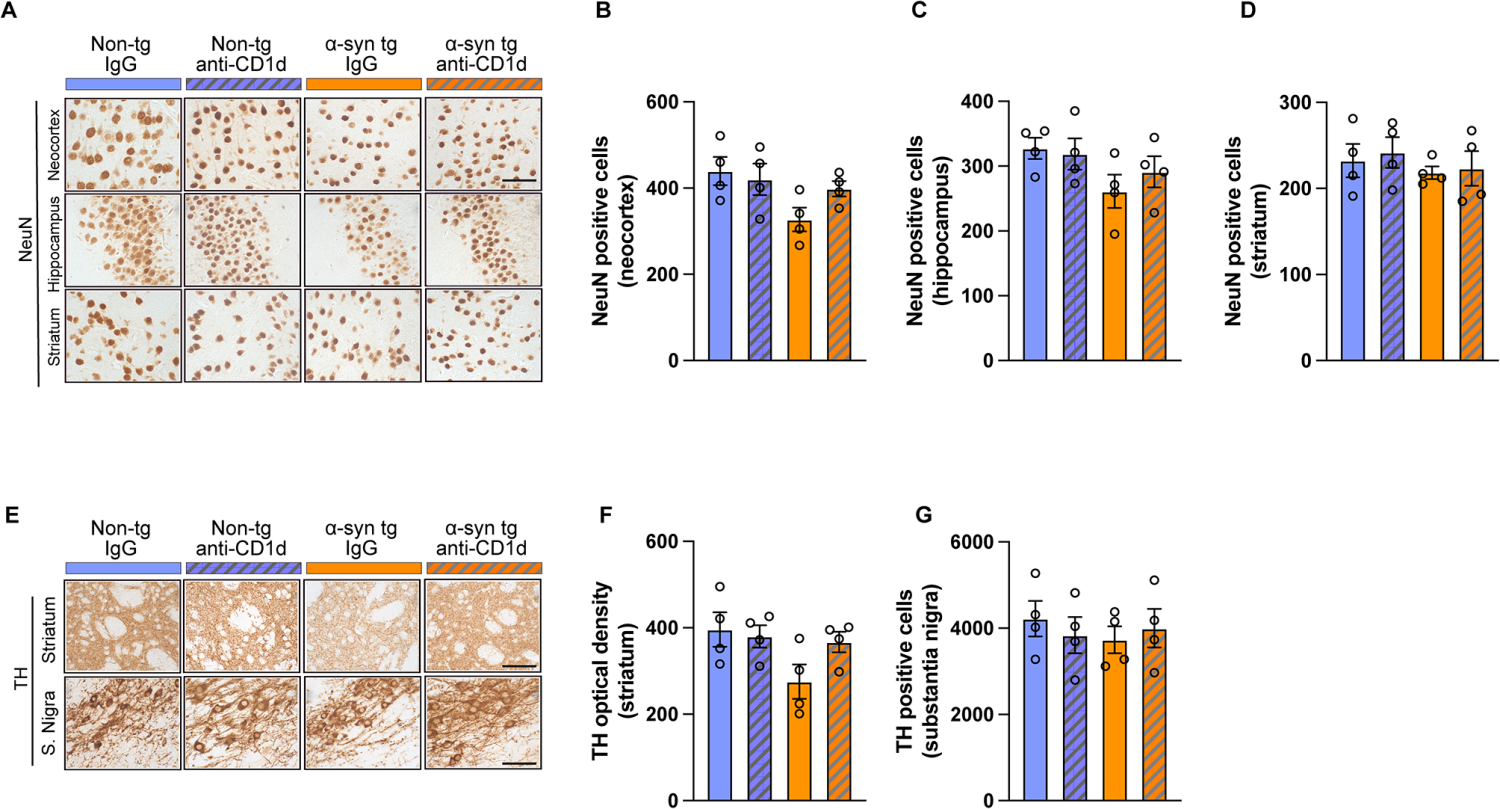
Immunohistochemical analysis of neurodegeneration in the brains of ⍺-synuclein transgenic mice treated with anti-CD1d. (**A**) Representative bright field light microscopy images from the neocortex, hippocampus, and striatum of non-tg and ⍺-syn tg mice treated with IgG or anti-CD1d immunostained with an antibody against NeuN (neuronal cell marker). (**B**–**D**) Computer based image analysis showing estimated numbers of NeuN immunoreactivity cells in the neocortex and hippocampus of ⍺-syn tg mice and normalized levels with anti-CD1d treatment. (**E**) Representative bright field light microscopy images from the striatum and S. Nigra of non-tg and ⍺-syn tg mice treated with IgG and anti-CD1d immunostained with an antibody against TH (dopaminergic marker). (**F**, **G**) Computer based image analysis showing levels of TH immunoreactivity fibers in the striatum of IgG treated ⍺-syn tg mice and effect with anti-CD1d therapy. Estimates of TH positive cells in the S. Nigra are comparable among the four groups. Scale bars = 50 μm for **A**, 40 μm for **E**. *n* = 4 mice per group. Statistical significance determined by two-way ANOVA with post-hoc Tukey

Further analysis of neuronal degeneration with an antibody against NeuN showed that the IgG treated α-syn tg mice showed a trend toward reduced numbers of cells in the neocortex and CA3 regions of the hippocampus (but not in the striatum) compared to IgG and anti-CD1d treated non-tg mice, while α-syn tg mice treated with the anti-CD1d antibody showed similar level as non-tg mice (Fig. 8A–D). There were no significant differences between four groups in any brain regions. Analysis of the dopaminergic neurons/terminals with an antibody against TH showed that the IgG treated α-syn tg mice showed a trend toward reduced density of TH fibers in the striatum compared to IgG and anti-CD1d treated non-tg mice, whereas α-syn tg mice treated with the anti-CD1d antibody showed comparable TH fiber density to non-tg mice (Fig. 8E, F). No differences were observed in TH neuronal cell numbers in the S. Nigra among the four groups (Fig. 8E, G).

**Fig. 9:**
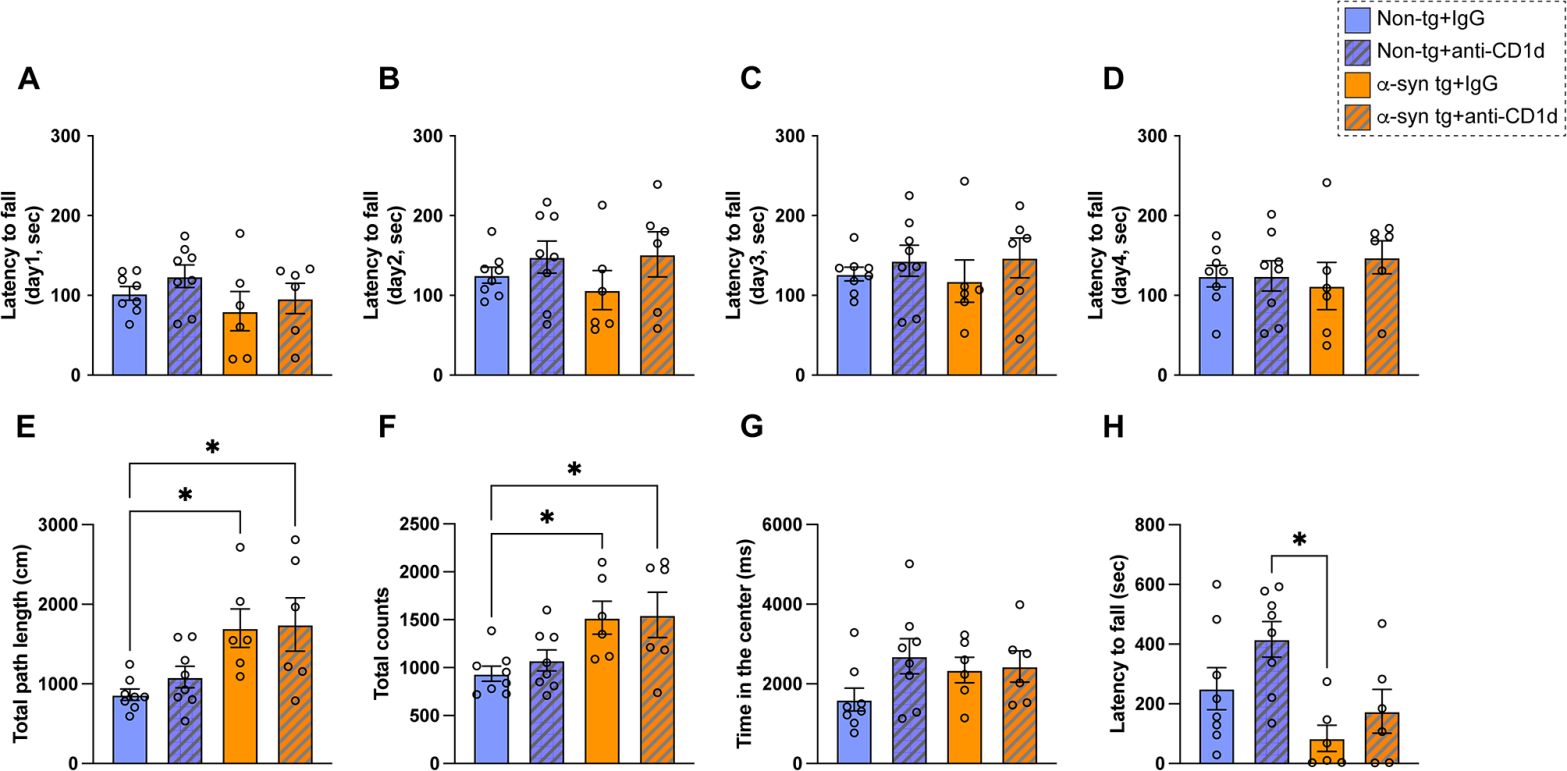
Behavioral analysis of ⍺-synuclein transgenic mice treated with anti-CD1d. (**A**–**D**) Rotarod motor testing in non-tg and ⍺-syn tg mice treated with IgG or anti-CD1d showing latency to fall over five trial per day for a total of four days. (**E**–**G**) Open field testing showing total path, total counts, and time in the center of the field in non-tg and ⍺-syn tg mice treated with IgG or anti-CD1d. (**H**) Wire hang test showing time expended suspending in the wire in day 1 and day 2 of the test in IgG and anti-CD1d treated non-tg and ⍺-syn tg mice. Statistical significance determined by two-way ANOVA with post-hoc Tukey; *, *p* ≤ 0.05

The rotarod test was used to test motor learning and coordination. Compared to IgG and anti-CD1d treated non-tg mice, the IgG treated α-syn tg mice showed a trend toward reduced motor learning and coordination, in contrast, α-syn tg mice with the anti-CD1d antibody treatment showed similar performance to the non-tg groups (Fig. 9A–D). In the open field test, although the IgG treated α-syn tg mice showed increased activity as reflected by the path length and total counts compared to IgG and anti-CD1d treated non-tg mice, treatment of the α-syn tg mice with the anti-CD1d antibody had no significant effects (Fig. 9E, F). No differences were detected among the four groups in the time spent in the center (Fig. 9G). In the wire hang test, compared to the anti-CD1d treated non-tg mice, the IgG treated α-syn tg mice showed profound shorter time to fall down, besides the anti-CD1d antibody treated α-syn tg mice showed a trend toward an improved performance compared to the IgG treated α-syn tg mice (Fig. 9H).

In conclusion, treatment of α-syn tg mice with the anti-CD1d antibody reduced neuroinflammation and ameliorated some aspects of neurodegeneration with some minimal functional effects.

## Discussion

There is increasing evidence that in addition to the contributions of innate immune responses [9, 13, 33–38] to AD and related dementias, infiltration by T cells, NKs and NKTs might also play an important role in the neuroinflammatory and neurodegenerative pathology [16, 39–41]. We have shown in agreement with other studies [17, 42, 43] that in DLB/PD and in models of synucleinopathy there is an age-dependent infiltration by NKTs into the brain [16]. Here we reasoned that given the potential contribution of the interactions between NKTs with glial cells in the CNS in models of DLB/PD, interfering with this process might ameliorate the neuroinflammatory response and neurodegeneration. This study showed that treatment with the anti-CD1d antibody decreased numbers of CD3/IFNγ-positive cells, consistent with NKTs, in the α-syn tg mice. This was accompanied by reduced CD1d-positive astrocytes, blunted cytokine response and decreased astrocytosis and microgliosis in the CNS of the α-syn tg mice. Moreover, anti-CD1d treatment ameliorated some aspects of the neurodegenerative pathology in the mouse model.

Consistent with our results that blockade of APC-NKT cell activation via systemic administration of anti-CD1d monoclonal antibodies is immunomodulatory, previous studies have shown that this approach prevented the injury-induced suppression of delayed hypersensitivity [44], accelerates wound closure [45], and that CD1d-specific single-domain antibodies can be used therapeutically in cancer and inflammatory disorders [46]. Other examples of potential therapeutic applications for neutralizing NKT cells with anti-CD1d antibodies includes asthma [47] and eosinophilic esophagitis [48]. CD1d is a 37 kDa glycoprotein expressed on the surface of various human APCs [49]. Previous studies have shown that neuroglial cells such as microglia and astrocytes might play a role as APCs in the CNS [14, 31]. In agreement with this possibility, we found that astrocytes display CD1d immunoreactivity and that in the IgG treated α-syn tg there was increased colocalization between GFAP and CD1d, while in animals treated with anti-CD1d this colocalization was reduced. This result might have different interpretations, one possibility being that the decreased trafficking of T cells (and interaction with APCs) results in a feedback loop with downregulation of CD1d expression, the other is that the anti-CD1d antibody triggered degradation of CD1d, or that the immunotherapy targeting CD1d had a masking effect on CD1d.

Here is worth mentioning that while antibodies against CD1d disrupts interactions between APCs and NKTs, treatment with antibodies NK1.1 reduces NK cells [50]. Typically, NKTs are a subset of CD1d-restricted T cells at the interface between the innate and adaptive immune system [51, 52]. NKT cells have characteristics of both conventional T cells and NK cells. Upon activation, NKT cells can kill target cells via activation of inflammatory responses [53]. In contrast, NK cells are a specialized type of lymphocytes with granules that can target and eliminate cancer cells and viruses [54]. In fact, it has been proposed that NK cells might also play a role in other neurodegenerative disorders [55] most prominently in AD [56, 57]. For example, in 3xTg-AD tg models of AD, reduction of NK cells (with anti-NK1.1) decreased neuroinflammation and behavioral decline without affecting levels of Amyloid beta pathology [50]. In contrast, CD1d-neutralizing antibodies did not significantly affect the cognitive function of 3xTg-AD mice, indicating that in models of AD most likely NK cells play a more central role in modulating neuroinflammatory responses while NKT cells might have a more discrete effect.

In models of DLB/PD, both NK cells and NKT cells might play an active role modulating neuroinflammation and neurodegeneration. In PD, NK cells have been proposed to have the ability to uptake and degrade extracellular α-syn aggregates [58] and while some studies suggest that NK cells might play a role in neuronal damage, others propose that they might be protective in models of PD [59]. Interestingly, in a preclinical model of PD, depletion of NK cells resulted in exacerbated motor deficits and increased phosphorylated α-syn deposits indicating a potential role of NK cells at clearing cytotoxic forms of α-syn [58]. The study by Earls et al. utilized an anti-NK1.1 monoclonal antibody (mAb) (PK136 clone) to deplete NK cells in tg mouse model (8- to 10-wk-old male and females) overexpressing human A53T α-syn mutant protein (M83 Tg) [58], while our study utilized the anti-CD1d antibody to interfere with the trafficking and interaction between NKTs and APCs in the CNS of tg mice overexpressing human wt α-syn (Line 61 model). Consistent with this possibility, we found that in the CNS there were decreased numbers of cells co-expressing CD3/IFNγ, and by flow cytometry we observed decreased numbers of NKTs in the spleen. Moreover, tg mice treated with IgG showed increased IFNγ expression by PCR, and anti-CD1d treatment normalized these levels. We did not detect significant changes in the levels of NKTs (or CD4/CD8 cells) in the liver or blood and, due to insufficient sample we were unable to detect NKTs in the brain by flow cytometry. These modest effects on neuroinflammation and neurodegeneration of the anti-CD1d treatment in our model might be related to the relative low dose of the immunotherapy, exposure, duration of the treatment, and number of animals. Future studies will be needed in a larger sample of animals, at higher doses and for a prolonged period comparing preventive vs. therapeutical interventions.

**Fig. 10:**
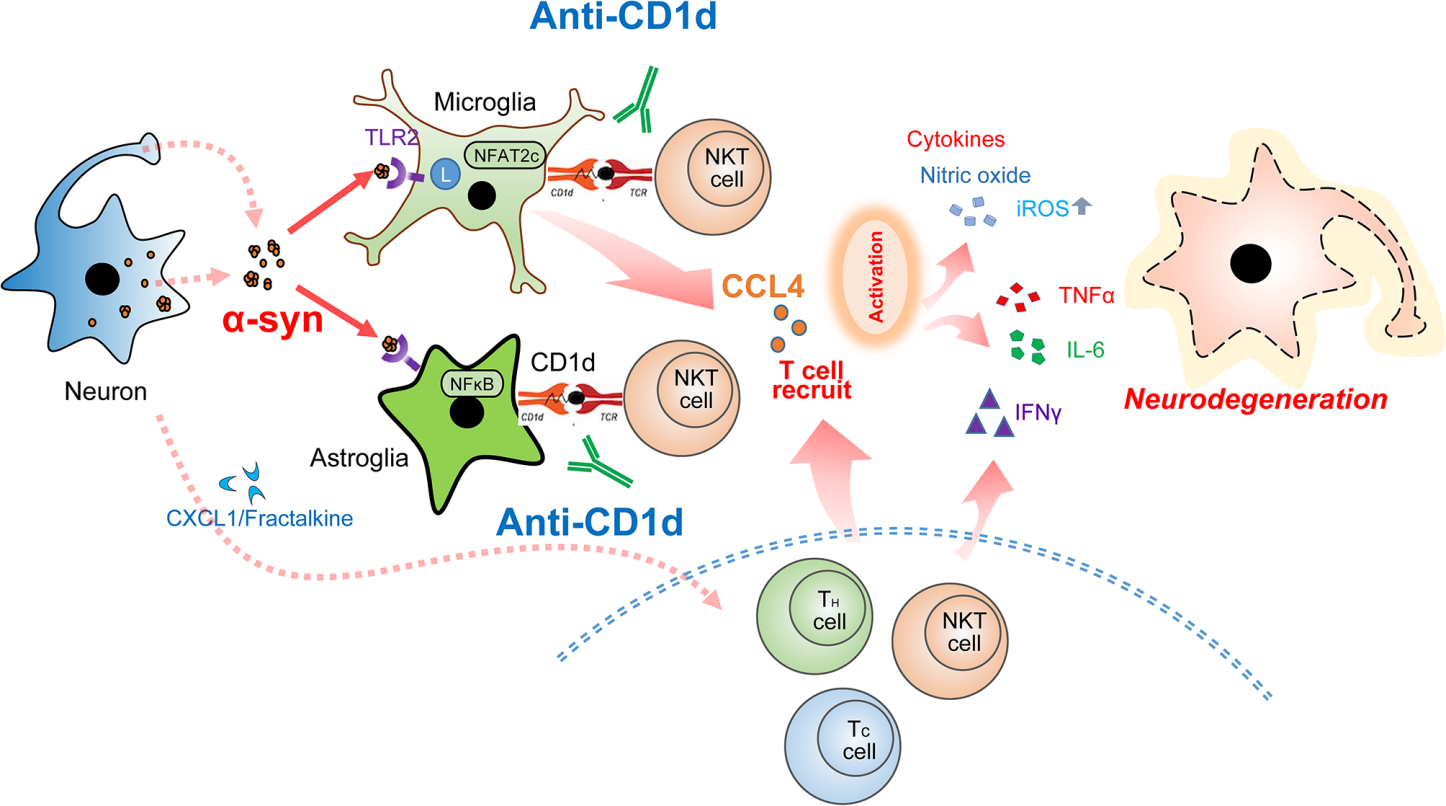
Diagrammatic representation of the possible mechanisms of action for the anti-CD1d treatment in synucleinopathy models. Extracellular α-syn aggregates targeting TLR’s activate microglia and astrocytes that might work as APCs in the CNS, in turn producing cytokines and chemokines (e.g.: CCL4) that attract migration of T cells and NKTs. Since neurotoxicity might occur when presenting lipid antigens by APCs CD1d- activate NKT cells, blocking the APC-NKT interaction with an anti-CD1d antibody reduces neuroinflammation and neurodegeneration by abrogating the production of toxic cytokines

Further supporting a potential effect of the anti-CD1d, we found that NKT cells were decreased and expression of IFNγ, CCL4, IL6 were reduced in the brains of the antibody-treated mice compared to IgG-treated α-syn tg mice. Remarkably, while in IgG-treated α-syn tg there was increased CCL4 levels, in animals treated with anti-CD1d the expression of CCL4 was comparable to controls. This inducible chemokine also known as macrophage inflammatory protein 1β (MIP1β) is as a chemoattractant for NKTs [60], monocytes [61] and various other immune cells in the site of inflamed or damaged tissues. CCL4, secreted extracellularly, binds to chemokine receptors (CCR1, CCR5) [62]. CCL4 is secreted from glial cells and astrocytes in the CNS and has been suggested to be involved in the progression of various brain diseases, including AD, multiple sclerosis, and ischemic brain disease [63–67]. CCL4 have also been extensively studied in the context of HIV infection since they compete with the HIV virus for binding to the same receptor [68] and levels of CCL4 are elevated in HIV neurocognitive disorders [69]. Our interest on CCL4 derives from its ability to attract NKTs among other immune cells, suggesting that in models of synucleinopathy the increased trafficking of some lymphocytic populations to the CNS might be in part mediated by CCL4 and that anti-CD1d immunotherapy might disrupt this process (Fig. 10). Other pathways in DLB and α-syn tg models for T cell migration into the brain proposes signaling via CXCR4-CXCL12 that results in interleukin-17 production and neurotoxicity [17].

In summary, extracellular α-syn aggregates targeting TLR’s activate microglia and astrocytes that might work as APCs in the CNS, in turn producing cytokines and chemokines (e.g.: CCL4) that attract migration of T cells and NKTs. The APCs CD1d-presented lipid antigens activate NKT cells through the interaction with TCR’s in NKTs resulting in the production of cytokines and neurotoxicity (Fig. 10). Thus, blocking the APC-NKT interaction with an anti-CD1d antibody reduced some aspects of neuroinflammation and neurodegeneration in models of DLB/PD and might be considered as an alternative immunotherapeutical approach for synucleinopathies of the aging population.

### Electronic supplementary material

Below is the link to the electronic supplementary material.

Supplementary Material 1

## Data Availability

No datasets were generated or analysed during the current study.

## Abbreviations

α-syn: α-synuclein
PD: Parkinson’s disease
DLB: Dementia with Lewy bodies
Tg: Transgenic
AD: Alzheimer’s disease
APP: Amyloid precursor protein
TLRs: Toll like receptors
Treg: Regulatory CD4 + T cells
MHC: Major histocompatibility complex
IFN: Interferon
NKT: natural killer T cells
CNS: Central nerve system
TCR: T-cell receptor
pff: preformed fibrils
