# Supplementary material for: Immunotherapy with an antibody against CD1d modulates neuroinflammation in an α-synuclein transgenic model of Lewy body like disease
Source: J Neuroinflammation. 2024 Apr 15;21:93. doi: 10.1186/s12974-024-03087-7 (PMC11017481; doi:10.1186/s12974-024-03087-7)
Supplement: Supplementary file 1 — Supplementary Material 1 [file 12974_2024_3087_MOESM1_ESM.docx]

Supplementary Information


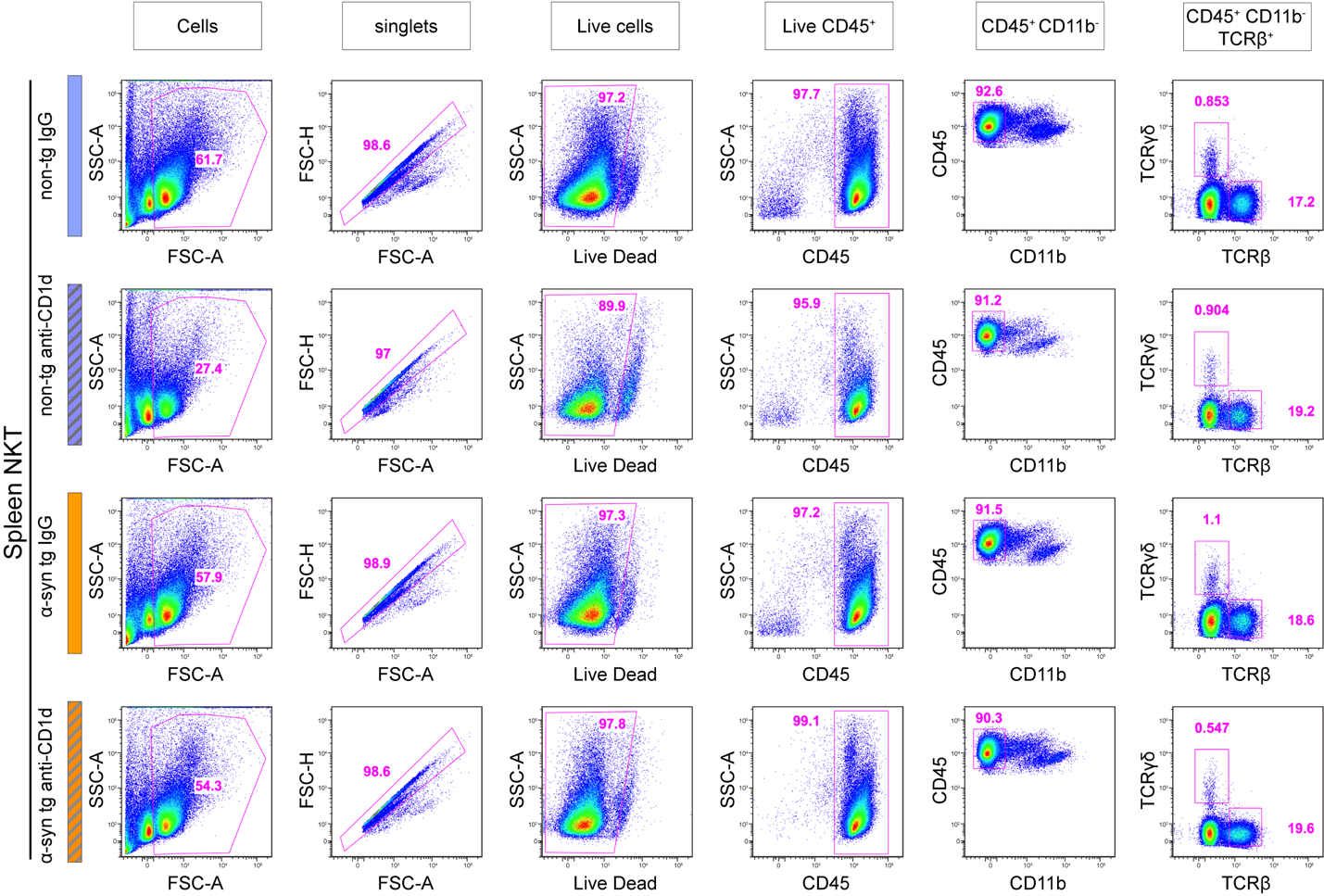


Figure S1. Complete gating strategies for the spleen. We represent whole cells, singlets, live cells, CD45^+^, CD45^+^/CD11b^-^, and CD45^+^/CD11b^-^/TCRβ for non-tg IgG, non-tg anti-CD1d, α-syn tg IgG, and α-syn tg anti-CD1d.


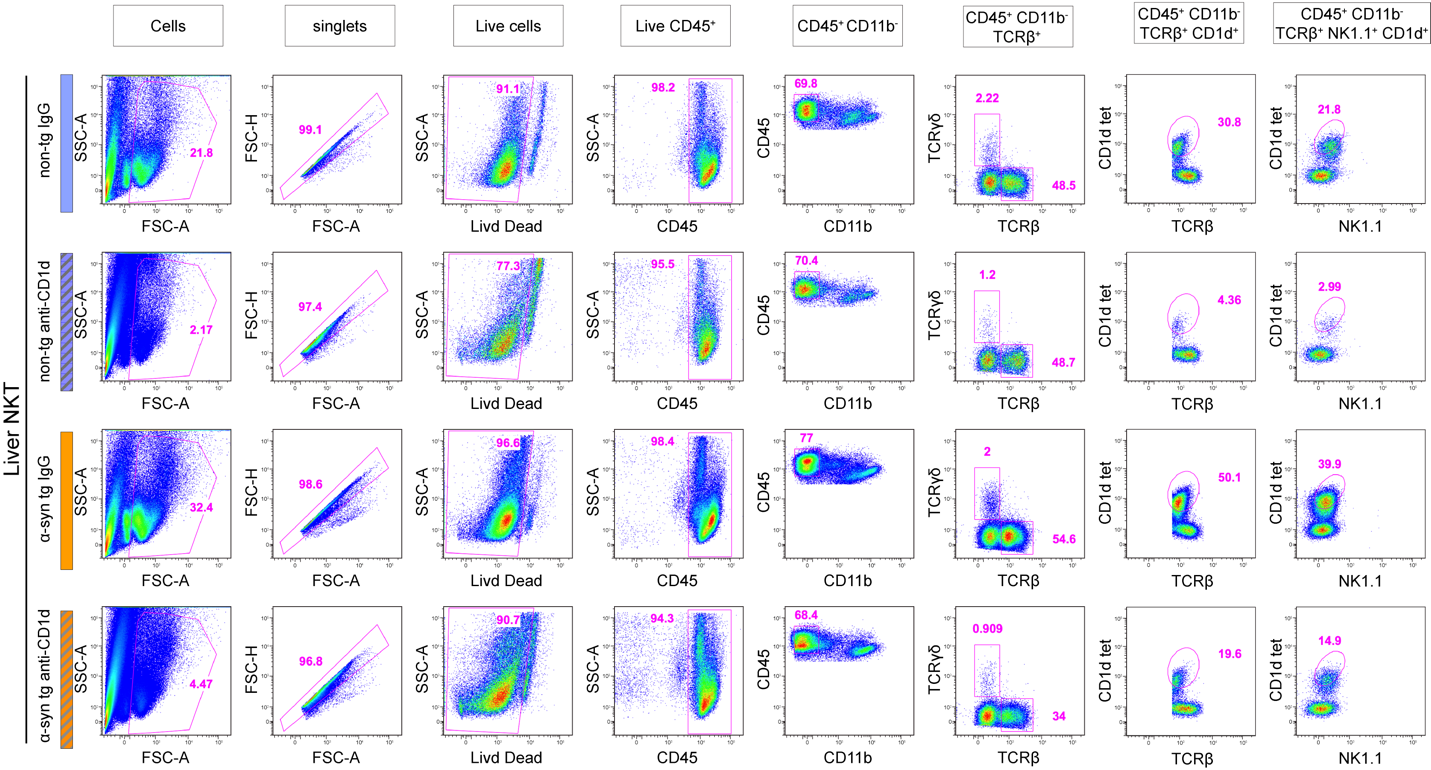


Figure S2. Complete gating strategies for liver. We represent whole cells, singlets, live cells, CD45^+^, CD45^+^/CD11b^-^, CD45^+^/CD11b^-^/TCRβ^+^, CD45^+^/CD11b^-^/TCRβ^+^/CD1d^+^ and CD45^+^/CD11b^+^/TCRβ^+^/NK1.1^+^/CD1d^+^ for non-tg IgG, non-tg anti-CD1d, α-syn tg IgG, and α-syn tg anti-CD1d.


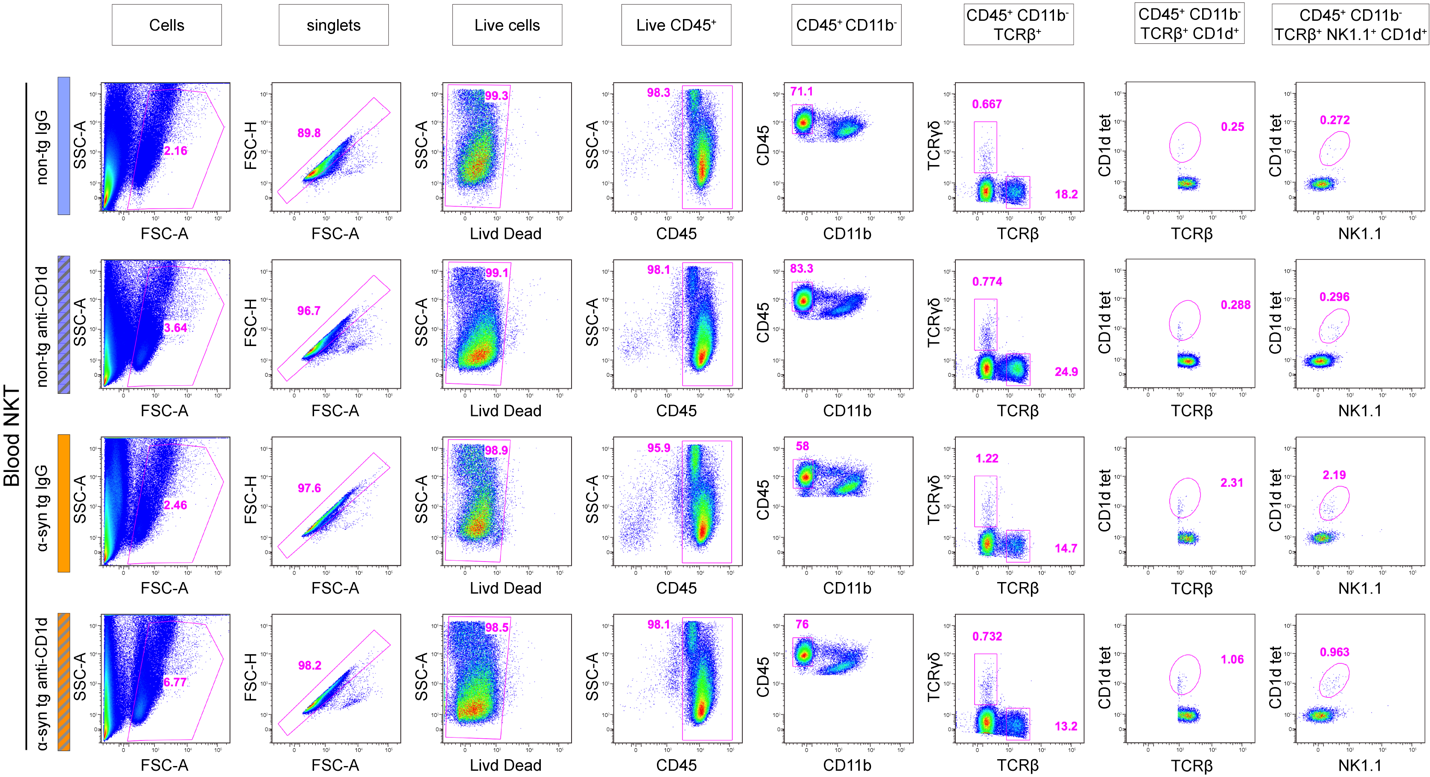


Figure S3. Complete gating strategies for blood. We represent whole cells, singlets, live cells, CD45^+^, CD45^+^/CD11b^-^, CD45^+^/CD11b^-^/TCRβ^+^, CD45^+^/CD11b^-^/TCRβ^+^/CD1d^+^ and CD45^+^/CD11b^+^/TCRβ^+^/NK1.1^+^/CD1d^+^ for non-tg IgG, non-tg anti-CD1d, α-syn tg IgG, and α-syn tg anti-CD1d.


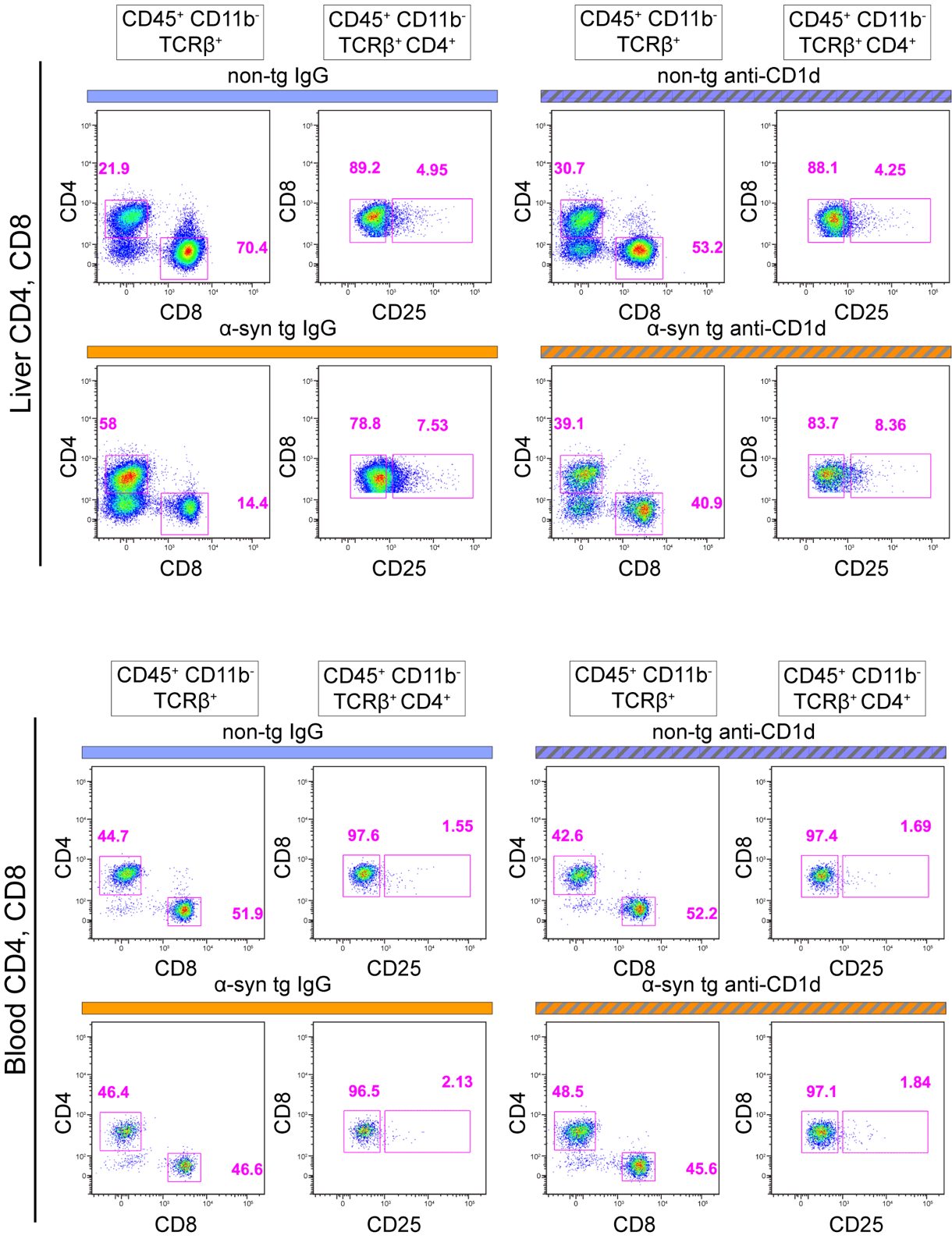


Figure S4. Representative flow cytometry plots of TCRβ^+^/CD45^+^ expression in liver and blood.
